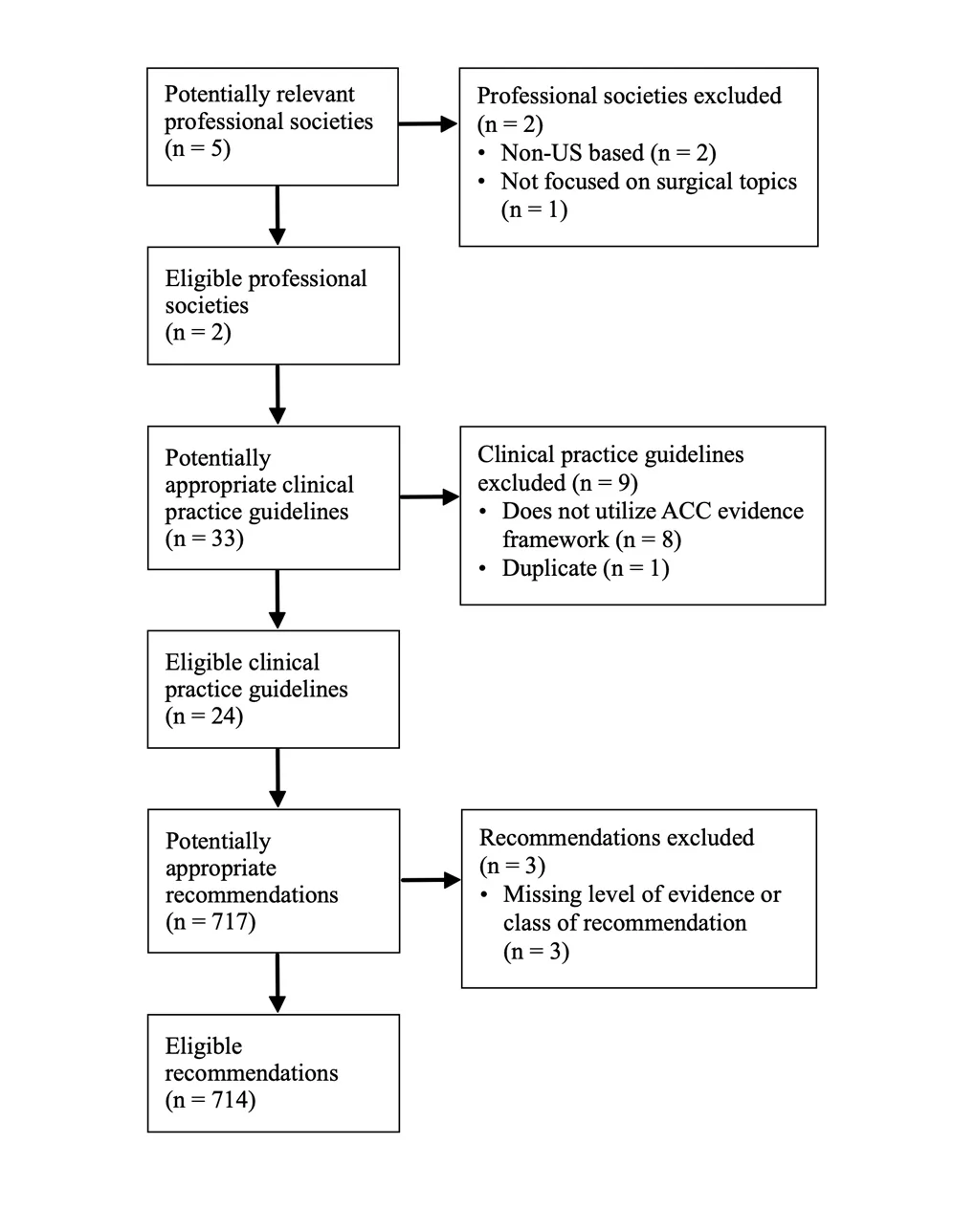# Assessing the Evidence Behind Guidelines in Cardiothoracic Surgery: A Systematic Review

**DOI:** 10.1016/j.atssr.2026.01.018

**Published:** 2026-02-26

**Authors:** Adrian Valderrama, Andrea Michelle Gochi, Amanda Soe, Elaine Liang, Alison Baskin, Jeffrey B. Velotta

**Affiliations:** 1 Department of Surgery, UCSF East Bay, Oakland, California; 2 UCSF School of Medicine, San Francisco, California; 3 Kaiser Permanente Bernard J. Tyson School of Medicine, Pasadena, California; 4 Department of Surgery, UCSF, San Francisco, California; 5 Department of Clinical Sciences, Kaiser Permanente Oakland Medical Center, Oakland, California; 6 Department of Surgery, Kaiser Permanente Bernard J. Tyson School of Medicine, Pasadena, California

## Abstract

**Background:**

Clinical practice guidelines are essential for guiding clinical decisions in cardiothoracic surgery. Despite this, the quality of evidence supporting cardiothoracic surgery guidelines has not been formally assessed. This systematic review characterizes the level of evidence underlying current cardiothoracic surgery guidelines.

**Methods:**

We extracted recommendations from US professional society guidelines that addressed cardiac or thoracic surgery and used the American College of Cardiology evidence framework. Recommendations were characterized by their level of evidence and assigned a phase of care by 2 independent raters. The primary outcome was the level of evidence across all guidelines, stratified by recommendation strength, phase of care, and disease process.

**Results:**

We identified 2 professional societies, American Association for Thoracic Surgery and The Society of Thoracic Surgeons, that published 24 guidelines meeting inclusion criteria. These contained 714 eligible recommendations; 52 (7%) had level A evidence, indicating support from multiple randomized controlled trials or 1 large randomized controlled trial. Of the recommendations classified as strong, 33 (10%) had level A evidence. Perioperative recommendations had the highest proportion of level A support (14%) compared with intraoperative (7%) and nonoperative (1%) recommendations. By disease process, level A evidence ranged from 44% in esophageal guidelines to 0% in coronary artery disease, aorta, and congenital heart disease guidelines.

**Conclusions:**

Few clinical practice guidelines in cardiothoracic surgery are supported by high-quality evidence. This highlights a significant evidence gap and the need to strengthen the rigor of evidence generation, particularly for surgical interventions.

**In Short**

▪ Only 7% of cardiothoracic guideline recommendations are supported by level A evidence.
▪ Several major domains—including aorta, coronary artery disease, lung transplant, and congenital heart disease—lack any recommendations based on level A evidence.
▪ Expanding randomized evidence in cardiothoracic surgery remains a critical need.

Clinical practice guidelines are developed to help translate evidence-based medicine into standardized recommendations to improve patient care. Guideline adherence can improve outcomes in multiple aspects of cardiothoracic care.^1^ Despite their broad influence, the evidentiary foundation for these recommendations varies, ranging from expert consensus to randomized controlled trials (RCTs).

To classify the strength of evidence supporting individual recommendations, guideline authors often employ formal grading frameworks. In cardiothoracic surgery, guidelines frequently implement the American College of Cardiology (ACC)/American Heart Association (AHA) model. This framework classifies recommendations on the basis of supporting evidence (Level of Evidence [LOE]) and strength (Class of Recommendation [COR]).^2^

RCTs are widely regarded as the highest standard of evidence; however, studies across multiple specialties have identified a paucity of RCT-based recommendations. In particular, recent reviews of guidelines in cardiology and general surgery found that just 8.5% and 11.5% of recommendations were supported by RCT data, respectively.^3^^,^^4^ This study evaluated the evidence supporting guideline recommendations in cardiothoracic surgery and aimed to identify potential areas in need of stronger evidence.

## Material and Methods

Societies were included if they were based in the United States, published cardiac or thoracic surgery guidelines, and used the ACC/AHA framework. Two professional societies met criteria: American Association for Thoracic Surgery and The Society of Thoracic Surgeons. We identified guidelines from their websites in February 2025. Guidelines were included if they implemented the ACC/AHA model. Those using alternative frameworks or expert opinion were excluded. Recommendations assigned both LOE and COR were included (Supplemental Figure).

For each recommendation, reviewers extracted the full text along with its LOE and COR. LOE assignments range from level A to level C. Level A reflects support from multiple RCTs; level B, from limited RCT data or high-quality nonrandomized studies; and level C, from limited data or expert opinion alone. The COR ranges from class I to class III, with class I indicating the strongest recommendations and class III, the weakest.

Two reviewers assessed each recommendation and assigned a phase of care classification—preoperative, perioperative, postoperative, postdischarge, or nonoperative—as defined by the American College of Surgeons.^5^ Reviewers worked independently and were blinded to each other's classifications. Discrepancies were resolved by third-party adjudication. Guidelines were also categorized by disease process into 4 overarching groups (adult cardiac, general thoracic, perioperative, and congenital) with further subdivision. For guidelines addressing multiple disease processes, individual recommendations were sorted accordingly.

The primary outcome was the distribution of the LOE underlying recommendations. Proportions of LOE A, B, and C were calculated and stratified by recommendation strength, phase of care, and disease process. Given a disproportionate contribution from the largest 3 guidelines, a sensitivity analysis was performed to assess their effect on the overall data set. A formal risk of bias assessment was not conducted because of the focus on clinical recommendations rather than on primary research. No statistical effect size measures were applied as analyses were descriptive in nature.

## Results

Of the 33 eligible guidelines, 24 met inclusion criteria. Of the 9 excluded, 7 relied on expert opinion, 1 used a non-ACC/AHA framework, and 1 was a duplicate. Included guidelines spanned 2006 to 2025, with most (79%) published after 2014 (Table 1). Collectively, these guidelines contained 717 recommendations, of which 3 lacked an assigned LOE or COR. The median number of recommendations per guideline was 16 (range, 4-162).

**Table 1:** Guideline Characteristics

Topic	No. of Recommendations	LOE A, %	Year
AATS			
Donor lung procurement	35	0	2025
Subsolid lung nodules	13	0	2024
Ebstein anomaly	12	0	2024
Early-stage non-small cell lung cancer	14	36	2023
Mechanical circulatory support in lung transplantation	36	0	2023
Tetralogy of Fallot	15	0	2023
Mechanical circulatory support	93	0.02	2020
Bicuspid aortic valve	29	0	2018
Surgical ablation for atrial fibrillation	10	5	2017
Empyema	39	0.05	2017
Anomalous aortic origin of coronary artery	15	0	2017
Infective endocarditis	80	0.01	2017
Stable ischemic heart disease	8	0	2014
STS			
Type B aortic dissection	17	0	2022
Perioperative blood management	37	24	2021
Anticoagulation during cardiopulmonary bypass	17	0	2018
Surgical treatment of atrial fibrillation	8	12	2017
Arterial conduits for coronary artery bypass	11	0	2016
Esophagus and gastroesophageal junction cancer	9	44	2014
Aortic valve and ascending aorta	162	8	2013
Protecting the infant brain during cardiac surgery	9	11	2012
Prophylaxis for atrial fibrillation associated with thoracic surgery	24	21	2011
Antibiotic prophylaxis in cardiac surgery, part II: antibiotic choice	17	24	2007
Antibiotic prophylaxis in cardiac surgery, part I: duration	4	0	2006

AATS, American Association for Thoracic Surgery; LOE, Level of Evidence; STS, The Society of Thoracic Surgeons.

Across all recommendations, LOE A supported 52 (7%) recommendations, LOE B supported 326 (46%), and LOE C supported 334 (47%). The median proportion of recommendations with LOE A in each guideline was 0.5%, with a range of 0% to 44%. Half (12/24) of all guidelines contained no LOE A recommendations.

Phase of care designations resulted in 74 (10%) nonoperative, 140 (20%) preoperative, 49 (7%) perioperative, 342 (48%) intraoperative, 57 (8%) postoperative, and 52 (7%) postdischarge classifications (Table 2). The highest proportion of LOE A support was for perioperative (14%) recommendations compared with intraoperative (7%) and nonoperative (1%) recommendations.

**Table 2:** Evidence Quality (LOE) by Phase of Care

Phase	No. of Recommendations	A	B	C
Nonoperative	74	1 (1.4)	40 (54)	33 (45)
Preoperative	140	11 (7.9)	57 (41)	72 (51)
Perioperative	49	7 (14)	22 (45)	20 (41)
Intraoperative	342	24 (7)	158 (46)	160 (47)
Postoperative	57	3 (5.3)	31 (54)	23 (40)
Postdischarge	52	6 (12)	18 (35)	28 (54)

Values are reported as number (percentage).

LOE, Level of Evidence; LOE A, multiple randomized controlled trials; LOE B, limited randomized controlled trials or high-quality nonrandomized studies; LOE C, limited data or expert opinion.

Based on strength (COR) classifications, 332 (47%) recommendations were strong (clear benefit), 245 (34%) moderate (may be reasonable), 93 (13%) weak (uncertain benefit), and 44 (6%) harmful (Table 3). Of the strong recommendations, 33 (10%) were supported by LOE A compared with 14 (6%) of moderate strength recommendations and 0% of weak recommendations.

**Table 3:** Evidence Quality (LOE) by Recommendation Strength (COR)

	A	B	C	Total
Strong (I)	33 (10)	161 (48)	138 (42)	332
(clear benefit)				
Moderate (IIa)	14 (5.7)	117 (48)	114 (47)	245
(may be reasonable)				
Weak (IIb)	0 (0)	29 (31)	64 (69)	93
(uncertain benefit)				
Harm (III)	5 (11)	19 (43)	20 (45)	44

Values are reported as number (percentage).

COR, Class of Recommendation; LOE, Level of Evidence; LOE A, multiple randomized controlled trials; LOE B, limited randomized controlled trials or high-quality nonrandomized studies; LOE C, limited data or expert opinion.

Stratification by overarching disease process showed that LOE A supported 29 (8%) adult cardiac, 11 (9%) general thoracic, 12 (6%) perioperative, and no congenital recommendations (Figure). Of the specific disease processes, the highest LOE A support was in esophageal (44%) and atrial fibrillation (26%) guidelines, whereas no LOE A supported aorta, coronary artery disease, and lung transplantation recommendations.

**Figure.**
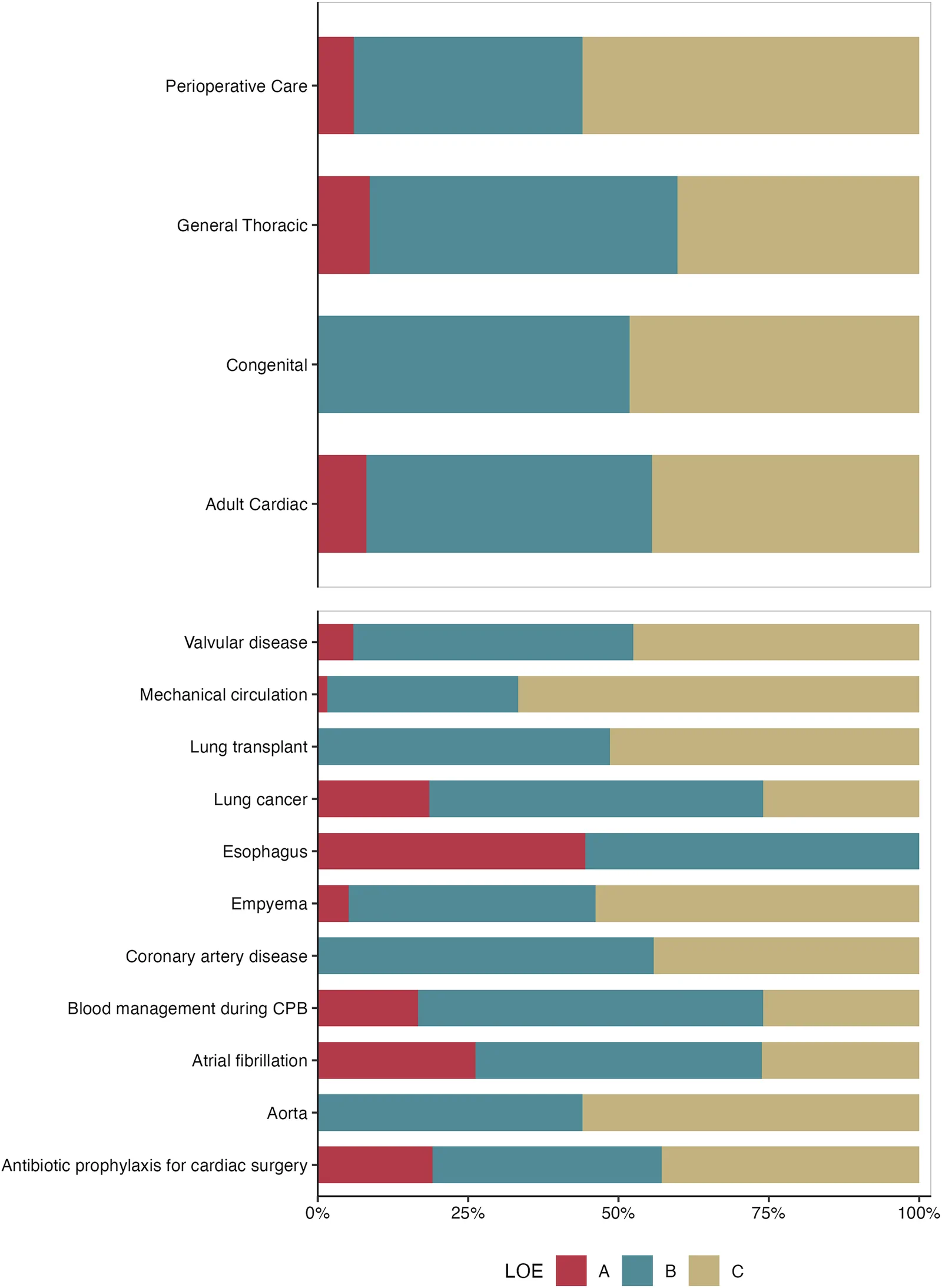
Evidence quality (LOE) by disease process. Level of evidence (LOE) by overarching and specific disease processes. (CPB, cardi opulmonary bypass.)

A sensitivity analysis was performed to assess for bias due to LOE distribution, given that the largest 3 guidelines accounted for 335 (47%) of all recommendations. Within this subgroup, 36 (9%) recommendations were supported by LOE A, 186 (49%) by LOE B, and 157 (41%) by LOE C, which reflected the overall data set.

## Comment

This study evaluated the quality of evidence supporting guidelines in cardiothoracic surgery. Of 24 guidelines, only 7% of recommendations and 10% of strong (class I) recommendations were backed by level A evidence (LOE A). These findings align with prior reviews demonstrating limited RCT data underlying clinical recommendations.^3^^,^^4^

Approximately half of all recommendations addressed intraoperative decision-making, with representatively low (7%) level A support. The challenges of conducting surgical RCTs are well established^6^; 1 analysis of 93 cardiothoracic RCTs (2008-2020) found frequent limitations in sample size, blinding, and assessment of operative technique.^7^^,^^8^ In contrast, perioperative recommendations were most often supported by LOE A (14%), likely reflecting their focus on protocol-driven therapies amenable to randomization.

Although recommendation strength is determined independently of LOE, the 2 were correlated. Strong recommendations were most often (10%) supported by LOE A compared with moderate (6%) and weak (0%) recommendations. Harmful recommendations had the most LOE A support (11%), which may reflect reclassification of prior guidance after new evidence or greater confidence in avoiding certain practices.

Multiple disease processes had no level A support, such as aorta and coronary artery disease. This may reflect a predominance of recommendations addressing operative approach (52% intraoperative). In contrast, those guidelines with the most level A evidence, esophagus and atrial fibrillation, contained many recommendations pertaining to medical rather than to surgical management; just 3% of recommendations focused on intraoperative care. We did not differentiate recommendations by procedure complexity, disease acuity, or rarity. These factors are likely to influence the feasibility of randomized evidence, and future work should stratify by these characteristics to identify granular barriers to conducting RCTs.

Our findings underscore the need for higher quality evidence in cardiothoracic surgery. Whereas previous studies have identified the overall low quality of evidence supporting clinical guidelines, our study provides domain-specific insight. Using multidimensional stratification, we demonstrate that the scarcity of randomized evidence is not only present but particularly pronounced for assessments of surgical interventions. Barriers to conducting surgical RCTs, including cost, equipoise, and logistics, warrant further study. Industry sponsorship plays a substantial role in evidence generation; most of the highest cited randomized trials indexed in Scopus received industry funding.^9^ Although we did not assess how funding sources affect guideline development, this remains an important area for future investigation.

In considering evidence quality, it is important to recognize that randomized evidence may not always be necessary or feasible to answer all clinical questions. Alternative study designs may offer a way to increase methodologic rigor; for example, pragmatic clinical trials are designed to evaluate interventions in real-world settings and offer flexibility that can reduce cost and operational burden.^10^ As such, pragmatic trials have been proposed as a middle ground between observational data and RCTs. As technology expands tools for evidence generation, it may be necessary to reconsider the paradigm of RCTs as the singular “gold standard” and to redefine grading systems accordingly.

This study has several limitations. First, it relies on the ACC/AHA framework; although standardized, the assignment of LOE and COR is subjective. Exclusive use of this schema required exclusion of guidelines using alternative frameworks, such as Grading of Recommendations Assessment, Development, and Evaluation (GRADE), thereby narrowing the range of included societies and recommendations. This decision maintained methodologic consistency and enabled direct comparison across documents. Second, the study is subject to sampling bias as 27% of guidelines were excluded. Seven of 9 exclusions were based on expert opinion; therefore, evidence quality may be overestimated. Finally, guideline size heterogeneity resulted in uneven weighting, although sensitivity analysis suggested that the largest guidelines were representative of the overall sample.

Only a small fraction of clinical recommendations in cardiothoracic surgery are supported by level A evidence. There remains an urgent need to develop innovative strategies for generating high-quality evidence, especially for surgical interventions.

## Figures and Tables

**Supplementary Figure 1:**